# EURISCO: The European search catalogue for plant genetic resources

**DOI:** 10.1093/nar/gkw755

**Published:** 2016-08-31

**Authors:** Stephan Weise, Markus Oppermann, Lorenzo Maggioni, Theo van Hintum, Helmut Knüpffer

**Affiliations:** 1 Leibniz Institute of Plant Genetics and Crop Plant Research (IPK) Gatersleben, Corrensstr. 3, 06466 Stadt Seeland, Germany; 2 Bioversity International, Via dei Tre Denari 472/a, 00057 Maccarese (Fiumicino), Rome, Italy; 3 Centre for Genetic Resources, The Netherlands (CGN), Wageningen University and Research Centre, P.O. Box 16, 6700 AA Wageningen, The Netherlands

## Abstract

The European Search Catalogue for Plant Genetic Resources, EURISCO, provides information about 1.8 million crop plant accessions preserved by almost 400 institutes in Europe and beyond. EURISCO is being maintained on behalf of the European Cooperative Programme for Plant Genetic Resources. It is based on a network of National Inventories of 43 member countries and represents an important effort for the preservation of world's agrobiological diversity by providing information about the large genetic diversity kept by the collaborating collections. Moreover, EURISCO also assists its member countries in fulfilling legal obligations and commitments, e.g. with respect to the International Treaty on Plant Genetic Resources, the Second Global Plan of Action for Plant Genetic Resources for Food and Agriculture of the United Nation's Food and Agriculture Organization, or the Convention on Biological Diversity. EURISCO is accessible at http://eurisco.ecpgr.org.

## INTRODUCTION

Crop plants are a major source for human and animal nutrition (1). Moreover, they play an important role for chemical and pharmaceutical industry and as renewable resources (2,3). To assure the future availability of the genetic diversity of crop plants and their wild relatives for use in plant breeding and research, this diversity needs to be preserved. Genebanks play an important role in the long-term conservation efforts of plant genetic resources for food and agriculture (PGRFA). However, their focus is not on conservation only. Genebanks also collect data about the material they conserve, thus allowing users to select the most appropriate material for use in their breeding or research programmes (4). An important component thereof is phenotypic characterisation of genebank accessions, i.e. collecting information about traits such as disease resistance, drought tolerance and yield components. These data are usually generated on selected material, resulting in non-orthogonal, highly incomplete data sets. Nevertheless, the analysis of these data allows meaningful results, e.g. the identification of promising new alleles (5). Around the world, there are about 1800 genebank collections conserving PGRFA. Thereof, about 625 collections are maintained in Europe comprising more than 2 million accessions (6).

The European Search Catalogue for Plant Genetic Resources (EURISCO) provides information about 1.8 million crop plant accessions comprising 6233 genera and 41 649 species. EURISCO was initially developed between 2001 and 2003 within the EU-funded project EPGRIS (European Plant Genetic Resources Information Infra-Structure) coordinated by the Centre for Genetic Resources, The Netherlands (CGN), and with the participation of the Czech Republic, France, Germany, Portugal, the International Plant Genetic Resources Institute (IPGRI, now Bioversity International) and the Nordic Gene Bank (NGB, now NordGen). In 2003, EURISCO became online accessible (7) and was hosted by Bioversity International, Rome, Italy, on behalf of the European Cooperative Programme for Plant Genetic Resources (ECPGR). In 2014, the Leibniz Institute of Plant Genetics and Crop Plant Research (IPK), Gatersleben, Germany, took over responsibility for the operation and development of EURISCO, as well as for the coordination of the EURISCO Network, still on behalf of ECPGR. The system was re-engineered completely and transferred to a new technological basis.

EURISCO is based on a network of National Focal Points (NFPs), who develop and maintain National Inventories (NIs) of the PGRFA holdings conserved in *ex situ* collections within their respective countries. The maintenance of most of these collections is supported by various management systems allowing provision of data to the respective NFPs who standardise the data in their NI, and regularly upload it to EURISCO, thus creating a complete overview of PGRFA in Europe.

## DATABASE DESCRIPTION

### Content

EURISCO contains both passport data and phenotypic information about plant genetic resources maintained in *ex situ* collections in Europe. Besides a research collection of the Nottingham Arabidopsis Stock Centre (http://arabidopsis.info/) comprising almost 670 000 *Arabidopsis thaliana* accessions, the major crops contained in EURISCO are wheat, barley and maize, respectively, which are among the top five major cereal grains produced worldwide (8). Table 1 gives an overview of the composition of EURISCO by plant species. The largest contributing National Inventories are those of the United Kingdom, Germany and the Russian Federation (Table 2).

**Table 1.** Content of the EURISCO database grouped by taxonomy showing the ten most frequent genera

Genus	Species	No. accs.	Total
*Arabidopsis*	*thaliana*^*a*^	669 381	669 587
	others	206	
*Triticum*	*aestivum*	104 985	147 055
(wheat)	*durum*	10 071	
	*turgidum*	8647	
	*monococcum*	3312	
	*spelta*	2760	
	others	17 280	
*Hordeum*	*vulgare*	84 087	105 289
(barley)	*spontaneum*	9746	
	others	11 456	
*Zea*	*mays*	61 799	61 932
(maize)	others	133	
*Phaseolus*	*vulgaris*	44 031	49 774
(garden bean)	*coccineus*	2829	
	others	2914	
*Solanum*	*lycopersicum*	18 338	44 400
(tomato, potato, eggplant, etc.)	*tuberosum*	13 857	
	*melongena*	2540	
	others	9665	
*Pisum*	*sativum*	26 517	29 735
(pea)	others	3218	
*Avena*	*sativa*	22 690	29 429
(oat)	*sterilis*	2123	
	*byzantina*	1045	
	others	3571	
*Vitis*	*vinifera*	24 941	28 819
(grape)	others	3878	
*Malus*	*domestica*	23 979	27 698
(apple)	others	3719	
others			649 034
		Total	1 842 752

^*a*^Model plant in life sciences research.

**Table 2.** The ten largest National Inventories providing data to EURISCO

Country	No. accs.	Percentage
United Kingdom	800 358	43.43%
Germany	174 362	9.46%
Russian Federation	123 430	6.70%
Ukraine	94 025	5.10%
Spain	76 984	4.18%
Poland	70 209	3.81%
Bulgaria	63 713	3.46%
Czech Republic	52 947	2.87%
Hungary	46 750	2.54%
Romania	46 039	2.50%
others	293 935	15.95%
Total	1 842 752	100.00%

**Figure 1.**
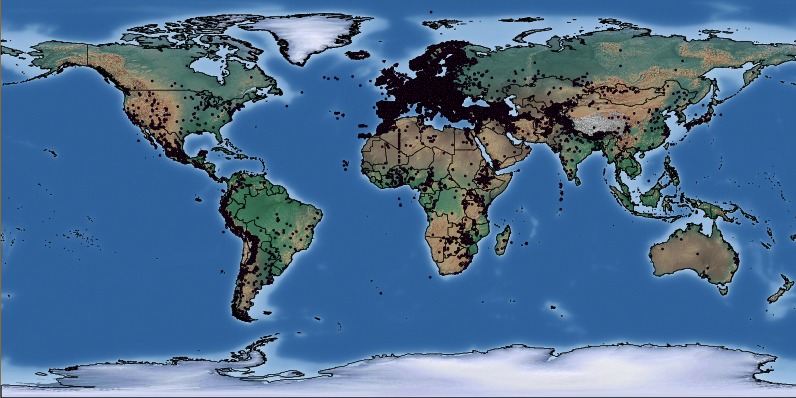
Collecting sites of accessions with geographical coordinates listed in EURISCO (map created by DIVA-GIS v7.5.0.0).

The participating PGRFA collections, due to their geographical location, focus on materials that can be maintained in the temperate climate zone. Twenty-eight countries of origin are represented by more than 10 000 accessions each, and 16 countries by more than 20 000 accessions, the five most frequent being Spain (66 327), Germany (55 349), the Russian Federation (49 781), USA (47 754) and Ukraine (43 617). The collecting sites of 188 454 EURISCO accessions having geographical coordinates are illustrated in Figure 1.

Additionally, EURISCO enables National Focal Points to label PGRFA accessions as part of AEGIS (A European Genebank Integrated System (6), http://aegis.cgiar.org/). AEGIS is an ECPGR initiative, aiming at improving the coordination of the conservation and management of PGRFA as well as the access to them, to ensure a safe long-term conservation (with common agreed standards) of genetically unique and important accessions. In order to reduce redundancy, the responsibilities for conservation are clearly defined. AEGIS is not a physical collection, but a virtual genebank. Changes of its composition are audited in EURISCO.

### Web interface

**Figure 2.**
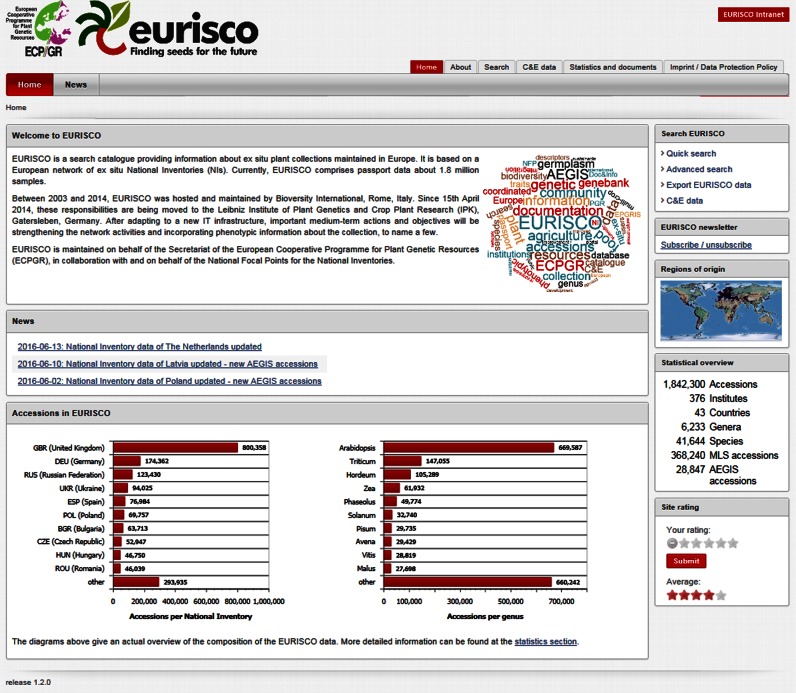
Overview screenshot of the EURISCO web interface.

The central entry point to EURISCO is the web interface (http://eurisco.ecpgr.org), shown in Figure 2.

**Figure 3.**
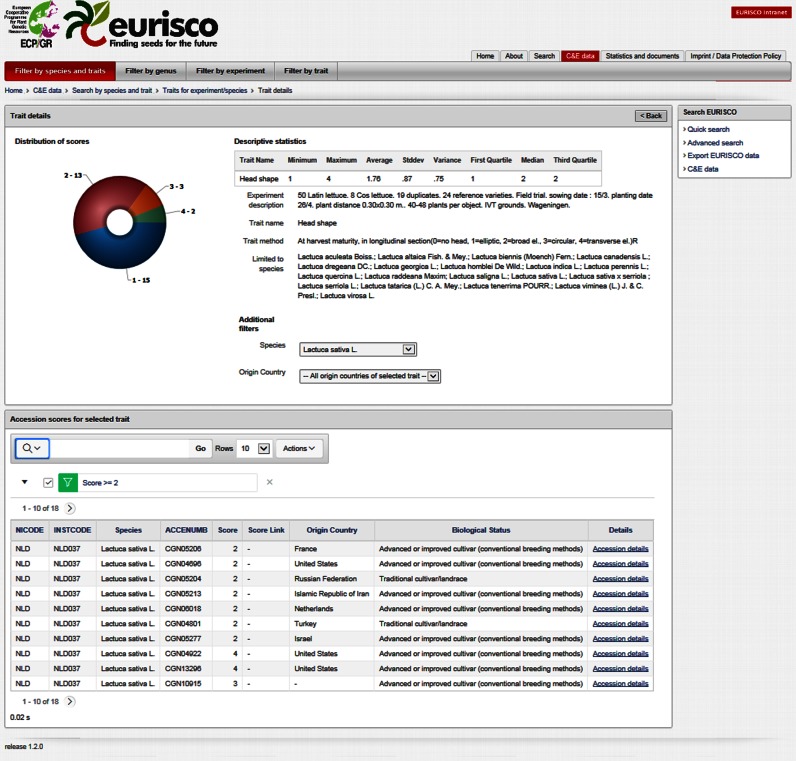
Filtering of phenotypic data using the EURISCO web interface.

The user interface provides different possibilities of retrieving information. Four standard searches are available, which enable the users to quickly search only those fields that are related to taxonomy, accession, biological status and collecting site, respectively. Additionally, an advanced search was implemented that allows to combine all available fields within a single search. Moreover, the available phenotypic data can also be searched and user-specific filter rules can be defined on the generated reports (Figure 3). All reports are available for download. In addition, various user-specific export functionalities including a full dump in MS-Access format are provided.

Furthermore, a variety of statistical reports as well as documents describing the background and the architecture of the EURISCO network are given. For disseminating information about EURISCO, a newsletter system using double opt-in was implemented.

For the development of the web interface, the Oracle Application Express (APEX, https://apex.oracle.com/) technology, version 5, was used. Further means of access, such as web service APIs, will be provided in the future.

### Database implementation

EURISCO was implemented on the basis of the Oracle relational database management system, version 12c (https://www.oracle.com/database/). The system comprises two parts, a so-called staging area for pre-processing and cleansing of data as well as database structures for the web front-end. The underlying database schema consists of 45 tables for the staging area, 40 tables for the front-end, 26 materialised views and 15 PL/SQL packages comprising 133 functions serving mainly for data quality assurance, user-specific download functionalities and reporting tasks.

## UPDATE PROCESS, QUALITY ASSURANCE AND CONTINUATION

The germplasm accessions listed in EURISCO are maintained by almost 400 institutes within the member countries. These institutes provide the data to their National Focal Points who compile the National Inventories of their respective countries and upload them to EURISCO, preferably at least once per year. For data exchange, standardised formats are used (FAO/Bioversity Multi-Crop Passport Descriptors format for passport data and a EURISCO-specific format for phenotypic data).

Via an intranet, data are uploaded by the National Focal Points into the staging area where they are extensively cleansed and checked for consistency. In this context, the correctness of scientific plant names (9) and the accuracy of geographic coordinates of collecting sites pose important challenges. The existing procedures allow the detection of typos in the taxonomy, while the geographical coordinates are automatically checked for compliance with the defined format, e.g. correct ranges of degrees, minutes, etc. There is room for additional developments in order to further improve the support to the data providers.

**Figure 4.**
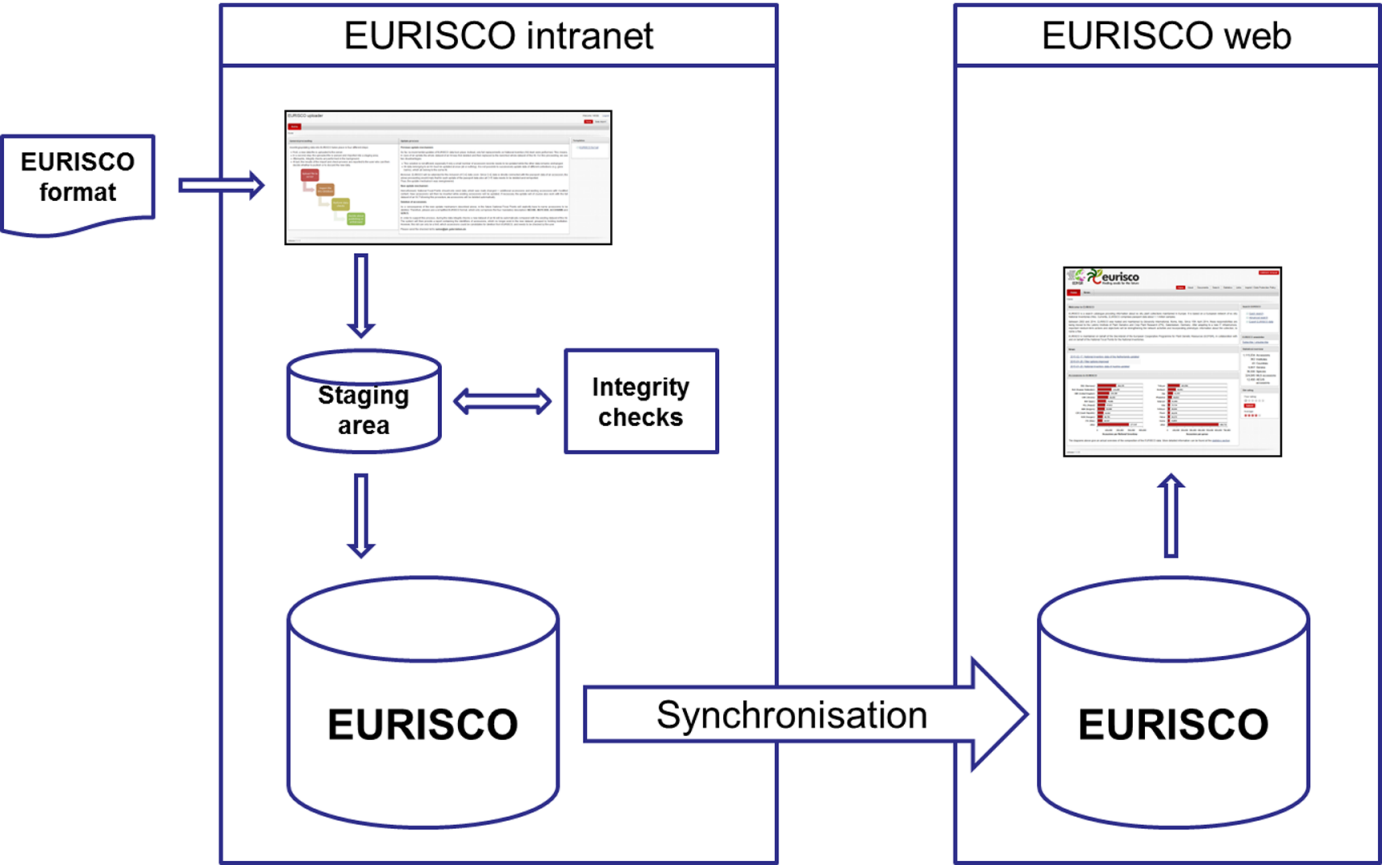
Architecture overview of the update process.

After approval by the data providers, the data are synchronised with the EURISCO web front-end (Figure 4).

Both data content and IT infrastructure are being improved continuously. The long-term maintenance of the EURISCO network will be ensured in the frame of the European Cooperative Programme for Plant Genetic Resources.

## APPLICATION

EURISCO serves a wide variety of applications in both preservation of biological diversity and crop plant research.

The central mission of EURISCO is to provide a one-stop-shop for information about the large genetic diversity existing in the collaborating collections for the scientific community and for plant breeders. In order to achieve the aim of sustainable breeding it is indispensable to mine the wealth of largely untapped genetic resources, such as crop wild relatives and old landraces. Here, EURISCO can provide important impulses since it maintains, amongst others, information about 233 905 crop wild relative accessions as well as about 252 130 landrace accessions.

Moreover, EURISCO also supports the coordination of efforts of the long-term maintenance of plant genetic resources among genebanks. It helps the member countries in fulfilling legal obligations and commitments, e.g. with regard to the International Treaty on Plant Genetic Resources (ITPGRFA, http://www.planttreaty.org/), the Second Global Plan of Action for Plant Genetic Resources for Food and Agriculture (Second GPA, http://www.fao.org/agriculture/crops/core-themes/theme/seeds-pgr/gpa/en/) of the United Nations Food and Agriculture Organization (FAO), or the Convention on Biological Diversity (CBD, https://www.cbd.int/), to name a few. All these agreements require the Parties to provide a transparent documentation of their respective PGRFA.

## DISCUSSION

EURISCO contains information about plant genetic resources for food and agriculture maintained in almost 400 institutions in Europe and beyond. It represents an important effort for the preservation and accessibility of world's biological diversity.

Besides the classical passport data, the system also provides phenotypic information about germplasm accessions. While the FAO/Bioversity Multi-Crop Passport Descriptors standard provides a well-established exchange format for passport data, there is no widely accepted format for phenotypic data existing (10). However, the scientific community is on the move. Initiatives such as Minimum Information about Plant Phenotyping Experiments (MIAPPE (11), http://www.miappe.org/) or CropOntology ((12), http://www.cropontology.org/) are emerging and could, in the long-run, lead to a significant improvement in the exchange and interpretation of phenotypic data.

Currently, EURISCO is limited to accessions maintained in *ex situ* collections. However, the inclusion of information about PGRFA maintained *in situ* is one of the development goals of the European Cooperative Programme for Plant Genetic Resources, which will be implemented in EURISCO in the future.

## CONCLUSION

EURISCO is an ongoing initiative that provides information about the majority of PGRFA accessions maintained in European collections. The vision for the system is in two directions: further extension of the database content in connection with increasing data quality, and improvement of the web interface.

Moreover, EURISCO will continue to cope with actual and upcoming topics within the PGRFA community, such as improved support for phenotypic data or unique identification of germplasm accessions.
